# Management of medical equipment in the Brazilian public health system (SUS), historical situation and the context of the pandemic COVID-19: a cut for lung ventilators

**DOI:** 10.1007/s12553-023-00752-4

**Published:** 2023-05-25

**Authors:** Fotini Santos Toscas, Eduardo Mario Dias, Maria Lídia Dias, Thiago Rodrigues Santos, Eduardo Jorge Valadares Oliveira

**Affiliations:** 1 Health Technologies Center for the Unified Health System, Health Institute, São Paulo, São Paulo, Brazil; 2 Polytechnic School, University of São Paulo, São Paulo, São Paulo, Brazil; 3 Secretariat of Science, Technology, Innovation and Strategic Health Supplies, Ministry of Health, Brasilia, Federal District, Brazil; 4 Strategic Health Technology Center, State University of Paraíba, Campina Grande, Paraíba, Brazil

**Keywords:** Equipment and supplies, Technology assessment, Biomedical, Medical equipment needs assessment, Technology management

## Abstract

**Purpose:**

The main objective of this paper is to analyze the Brazilian Ministry of Health (MoH) efforts in the management of medical equipment, with a specific approach for lung ventilators in the pandemic scenario of COVID-19.

**Methods:**

The methodology included a review of the normative framework and literature on technological management and research on the database of the Ministry of Health.

**Results:**

As a promoter for acquiring medical equipment, the MoH role is highlighted and added to this competence; its function as the coordinator of the National Policy on Health Technology Management (PNGTS). According to the PNGTS the MoH has to support health managers in the implementing, monitoring, and maintaining health technologies. The scenario of lung ventilators in the pandemic was discussed, with research to verify demands, offers, installed capacity, and investments. In less than one year, the Ministry of Health acquired several pulmonary ventilators, 8.55 times greater than the annual averages of equipment acquired from 2016 to 2019. So far, there is still no maintenance plans or strategy of management for that equipment, especially in a post-pandemic scenario. Conclusion: It is possible to conclude that the Ministry of Health needs to improve health technology management systems. On the scale of the Policy, it is necessary to commit to permanent and long-term actions to ensure sustainability and reduce the technological vulnerabilities of the SUS.

## Introduction

The Brazilian Ministry of Health (MoH) has historically made efforts to adapt its installed infrastructures through investments based on Programs to Strengthen the Unified Health System (SUS). One of the MoH financial efforts is "Action 8535—Structuring of Specialized Health Care Units" [1], which in 2019 had a budgetary commitment of nearly US$ 470 million and in 2020 about US$ 327 million [2].

Through these investments to public and private non-profit institutions, linked to SUS, the MoH substantially fosters the market for medical-hospital equipment with more than US$ 140 million per year, intended exclusively to purchase medical-hospital equipment and materials permanents [3]. In 2018, the MoH budget for acquiring such equipment jumped to 187 million, and in 2020, during the coronavirus pandemic, the value exceeded US$ 234 million [1–4].

The MoH's first formal initiative on medical-hospital equipment management was in1990. The MoH created the Dental-Medical-Hospital Equipment Program (PROEQUIPO). This program aimed to contribute so that the health system had, at all levels (municipal, state, and federal levels), dental-medical-hospital equipment addressed to international technical standards regarding safety and quality and qualified human resources to manage this entire installed park.

This initiative hoped to avoid operators' and patients' risks, wasted financial resources, and early scrapping of equipment [5].

Between 1992 and 1996, the Program allowed:

i) the creation of a specialization course in the area of management and maintenance of medical equipment for professionals of different levels of education (basic, medium, and higher levels);
ii) the development and internalization of international technical standards for the certification and improvement of the quality of the manufacture of electromedical equipment;
iii) the creation of electromedical equipment testing laboratories for certification procedures;
iv) the reformulation of the standard on hospital construction (Ministerial Order MoH nº 400/1977), which later originated the Ministerial Order MoH nº 1884/1994;
v) the development of technical manuals to guide health professionals in the areas of safety, planning, and dimensioning of hospital environments;
vi) the development of standards and ordinances by the Ministry of Health to improve the quality and safety of medical equipment;
vii) and the mandatory registration of medical equipment and articles sold in the country [6].

In January 1997, the MoH published the operative manual for the SUS Reorganization and Reinforcement Project (REFORSUS). The World Bank—WB financed the Project to recover the country's health network infrastructure by purchasing medical equipment, renovation, expansion, and conclusion of health institutions infrastructure, and actions to improve the management of services. These initiatives resulted in a considerable increase in the value of the technological park of SUS healthcare establishments, which in 2015 arrived at the level of 63,626 institutions involving around 17.5 million dollars in investments [7, 8].

Within the scope of REFORSUS, MoH carried out several actions and initiatives to prepare SUS technical staff in the management of health technologies. In 2002 the MoH made a public call to finance a distance learning project related to the training of national human resources on medical and hospital equipment management. This action was named the Medical- Hospital Equipment Maintenance Management (GEMA).

In mid-2001, the Brazilian National Health Surveillance Agency (Anvisa) instituted the Sentinela Network intending to be an active observatory of the performance and safety of regularly used health products: medicines, IVD, orthoses, prostheses, equipment, and medical- hospital materials, sanitizing, blood and its components. Among its specific objectives, the following stand out: i) obtain quality information on adverse events and technical complaints related to products under surveillance in the post-use / post-marketing period—VIGIPOS, to support decision-making by the National Surveillance System Sanitary (SNVS); ii) contribute to the improvement of risk management in health services; iii) develop and support studies of interests to the Brazilian Public Health System [9]. The intention is for the Network to continue to improve and consolidate itself as a reference for VIGIPOS, with quality gains and revision in its surveillance and risk management work processes.

In 2007, the Support System for the Elaboration of Health Investment Projects (SOMASUS) was created, which is a computerized system with free access, developed by the Executive Secretariat of the MoH, to assist states, municipalities, and institutions of the SUS in the elaboration of infrastructure investment projects in a more qualified way. The information provided by SOMASUS includes layouts of the environments of health establishments, their respective technical characteristics, and comprehensive content to support dimensioning, acquisition, installation, and operation of medical assistance equipment. The development of SOMASUS was based on the premise that establishing standards is essential for planning health investments. Therefore, the available content is based on the parameters from SUS care coverage, ministerial ordinances, technical standards, and Anvisa collegiate directive resolutions [10].

In early 2008, Ordinance nº 375/GM/MS was published, which instituted the National Program for Qualification, Production, and Innovation in Equipment and Materials for Use in Health at the Industrial Health Complex. The General Coordination of Equipment and Materials in Health (CGMES) within the structure of the Department of the Industrial Health Complex(DECIIS) of the Secretariat of Science, Technology and Strategic Inputs (SCTIE) [11].

In May 2019, through Decree nº 9,795, DECIIS and, consequently, CGEMS were extinguished [12]. In this way, the segment was left without a specialized technical area to respond to the qualifications, production, and innovation in medical and hospital equipment.

Already in 2010, the National Health Technology Management Policy (PNGTS) was published, which is the guiding instrument for the actors involved in the management of the assessment, incorporation, dissemination, management of the use and withdrawal of technologies in the Unified Health System. PNGTS establishes that it is up to the MoH to support managers in the implementation of technologies and in their monitoring and maintenance after incorporation [13].

In 2011, the MoH instituted the Health Network Quality Improvement and Training Project (QualiSUS-Rede), with an intervention proposal to support the organization of regionalized health care networks in Brazil. It was a cooperation between the World Bank and the Ministry of Health, which aims to add to the ongoing efforts to consolidate SUS. There has been a quantitative and qualitative leap in providing health technologies to the population in recent years. This new reality has demanded from health managers an increasing effort to train professionals to manage medical-assistance equipment to optimize the use of public resources and expand the population's access to this equipment [14].

Also, in 2011, Law 12,401 was published, and it provides rules for therapeutic assistance and the incorporation of health technology within the scope of SUS. Decree nº 7,646 provides for the National Commission for the Incorporation of Technologies in the Unified Health System (CONITEC) and the administrative process for incorporating exclusion and altering health technologies SUS [15].

In 2013, through Ordinance GM/MS nº 3134, the National List of Equipment and Permanent Materials financed by SUS (RENEM) was created to manage the financeable items for SUS and standardize their nomenclatures, allowing their effective management. RENEM items are made available to register project proposals according to the type of Health Care Establishment and their respective environments, organized by the Equipment and Materials Information and Management System (SIGEM) [16].

At the end of 2014, Ordinance nº 2,531/GM/MS was published, which redefined the guidelines and criteria for defining the list of strategic products for SUS and the establishment of Partnerships for Productive Development (PDP) [17].

## Methods

We carried out a literature search for normative account and survey of descriptive and exploratory data on the management of medical and hospital equipment within the scope of the MoH: i) National Register of Health Establishments (CNES) of the Department of Informatics of SUS (DATASUS), ii) and the System of Management of Equipment and Permanent Financeable Materials for SUS (SIGEM) of the National Health Fund (FNS). CNES provides information on the registration of Health Establishments regarding the physical area, human resources, equipment, and outpatient and hospital services. SIGEM, on the other hand, is the system that makes the information on financial equipment available to SUS. Data search was also carried out in the Federal Government's Integrated Financial Administration System (SIAFI), an accounting system to carry out all the processing, control, and financial, patrimonial, and accounting executions of the Brazilian federal government. Finally, we consulted the database on the ANVISA website to obtain information on the health records of lung ventilators.

We also made consultations in the MoH website database dedicated to coronavirus data, https://covid.saude.gov.br/, containing information on the pandemic's Brazilian response. The original search was carried out between March 2020 and December 2020.

In addition to research of an applied nature with a qualitative and quantitative approach, it is considered the authors' practical experiences in managing medical-hospital equipment and the health industrial complex.

## Results

The lung ventilator is a device used in cases of respiratory failure, with the function of pumping oxygen-enriched air into the lungs. In the SIGEM there is the item with the nomenclature Pressometric and Volumetric Lung Ventilator with a suggested value: US$ 11.440,7749 [18].

In consultation with SIGEM, in March 2020, approvals for decentralized purchases of lung ventilators in the last four years were verified, Table 1. It should be noted that the approvals only mean that the project proposals submitted by the Institutions obtained technical opinions on merits and favorable equipment. However, other imperative phases are necessary to carry out the transfer authorization contract and make the investment project feasible in acquiring medical and hospital equipment—modality of transfers of resources for decentralized purchase.

**Table 1:** Lung ventilators approved in the previous years

**Year**	**Quantitative**	**Value**
2016	1.995	U$ 24,571,803.28
2017	2.356	U$ 30,406,633.96
2018	1.904	U$ 22,948,774.71
2019	1.344	U$ 21,105,110.39

Source: extracted from SIGEM. Elaboration of the authors—Based on average annual dollar

Checking the approval data in the last year, 2019, we can see that 76% of the approved items have specification and value (US$ 12,000.00) suggested by the Ministry of Health, which indicates that the settings definitions are mostly standardized.

In consultation with the National Register of Health Establishments (CNES-DATASUS) [19] in March 2020, 65,205 existing lung ventilators and 43,758 in use in SUS were checked, Table 2.

**Table 2:** Installed Capacity CNES-DATASUS lung ventilators

**Code**	**Equipment**	**Existing**	**In Use**	**Existing SUS**	**In Use SUS**
64	Lung Ventilators	65,205	61,588	46,691	43,758

Source: extracted from CNES-DATASUS http://cnes2.datasus.gov.br/. Elaboration of the authors

In a consultation carried out (March 2020), 60 records of volumetric lung ventilators were found on the Anvisa website [20]. Of the 20 existing suppliers, 7 (seven) are national manufacturers and 13 (thirteen) international manufacturers.

In the paper entitled "Study of Medical-Assistance Equipment, Orthotics, Prosthetics, and Special Materials in the Unified Health System (SUS) in Brazil," published in March 2019, the authors intended to consolidate the strategic and priority products in a single list through the analyses of computerized databases, publications, and consultations with the technical and final areas of the Ministry of Health. In addition to crossing the information with the previous publications on the list of strategic products, assistance demands, incidences and ABC curve, judicialization, and recent incorporation in SUS. The study considered all the criteria with the same relevance and without weighting. The lung ventilator appeared in four out of five possible criteria, showing its relevance to the services and the health system [21]. The lung ventilator was initially included in the MoH list of strategic products in 2008, and in 2013 its importance was reiterated, respectively, Ordinance GM nº 978/2008 and nº 3,089/2013.

With the establishment of a pandemic of international importance due to the new human coronavirus, MoH took measures to deal with the public health emergency, mainly to provide the historical care demand for lung ventilators.

Interministerial efforts through a workgroup coordinated by the Special Secretariat for Productivity and Employment of the Ministry of Economic (MoE), with broad support and participations from the business community, the productive sector, and financial agents gathered energy to supply the necessary demand of SUS in lung ventilators. Highlighting that this happened almost a year after the extinction of CGEMS and DECIIS within the MoH, remembering that one of its competencies was coordinating activities for qualification, productions, and innovation in medical and hospital equipment.

In addition to the extinction of DECIIS, the MoH extinguished the Executive Group for the Industrial Health Complex—GECIS. This Forum, coordinated by the MoH, was a formal representation of fourteen bodies or entities of the Federal Public Administration, involving the decision-making nucleus of national development policy (State Department, Formal Ministry of Finance and Planning; Ministry of Foreign Affairs; Ministry of Development, Industry and Foreign Trade, and Ministry of Science, Technology, and Innovation), national development and regulation agencies and ST&I institutions. With this Forum, the SUS National Directorate began to coordinate, in an unprecedented way, a priority policy for industrial, technological, and innovation development, placing itself as an instance of articulation of the economic, industrial, and technological area to meet the imperatives of SUS [22].

Among the actions carried out by the interministerial group to supply the care demand for lung ventilators, the following stand out:

- a) *Productive escalation:* First, MoH specified the demand and criteria for ventilatory therapy for patients affected by COVID-19. Then, the MoH stimulated partnerships for large-scale product developments, arrangements for the production of critical components, and all the logistics necessary for the national companies that produce lung ventilators to make an exponential leap in their production to supply the centralized acquisition of MoH. Table 3 presents the data from the centralized purchasing contracts carried out from this initiative, a total of 16,252 lung ventilators.

**Table 3:** Contracts for centralized purchase of ventilators

**Agreement**	**Company**	**Qtd**	**Total Contract Value**	**Amount Paid**
120/2020	Magnamed	6.500	U$ 75,538,735.36	U$ 60,120,147.54
137/2020	Intermed	4.300	U$ 60,421,545.67	U$ 73,009,367.68
145/2020	KTK	3300	U$ 18,266,978.92	U$ 19,057,377.05
151/2020	Leistung	1202	U$ 16,870,491.80	U$ 20,383,372.37
179/2020	WEG DRIVES	950	U$ 13,348,946.14	U$ 7,025,761.12
Total		16.252	**U$ 184,446,697.89**	**U$ 179,596,025.76**

Source: extracted from https://antigo.saude.gov.br/contratos-coronavirus and consultation SIAFI Government Action: 21C0: FACING THE HEALTH EMERGENCY PUBLICA DE IMPORTANCIA. Elaboration of the authors

- b) *More Maintenance Initiative Lung Ventilators:* The Initiative + maintenance was carried out through a voluntary network to maintain unused lung ventilators, estimated at 3,000 lungs ventilators. The voluntary network was formed by the National Service for Industrial Learning (SENAI), ArcelorMittal, BMW Group, Fiat Chrysler Automóveis (FCA), Globo Comunicação e Participações, Ford, Oswaldo Cruz Foundation, General Motors, Honda, Hyundai Motor Brazil, Votorantim Institute, Technological Research Institute (IPT) and POLI-USP, Jaguar Land Rover, Mercedes-Benz do Brazil, Moto Honda, Petrobras, Renault, Scania, Todos pela Saúde (Itaú), Toyota, Troller, Usiminas, Vale, Volkswagen do Brazil and Volvo do Brazil. Supported by the Ministry of Health, the Ministry of Economy, the Brazilian Agency for Industrial Development (ABDI), and the Brazilian Association of Clinical Engineering (ABEClin). It had 40 points to receive the equipment. It involved more than 500 engineers and trained technical volunteers. More than 2,500 lung ventilators were recovered [23, 24].

Regarding the national lung ventilator manufacturers, it is essential to register the investments and government subsidies that allowed the country to have the productive capacity to respond to the health emergency. Among those manufacturers, we highlight the history of KTK Takaoka, specifically the young medical student Kentaro Takaoka, who, with subsidies from USP's Institute of Technological Research (IPT). He set up a plant in the building of the Clinical Hospital of the Faculty of Medicine. He developed reduced dimensions equipment capable of performing controlled artificial ventilation, winning in 2005 Finep (Brazilian Innovation Agency) Inventor Innovator trophy [25].

Also, there was support for the Magnamed company, founded in 2005. This company was one of the first companies invested by the Center for Innovation, Entrepreneurship, and Technology (Criatec), in 2014 it received from BNDES, through PSI—Innovation (Investment Support Program), credit for a new lung ventilator development project and in 2015 support from FINEP—Innovation and Research.

As Marcelo Miterhof, an economist at BNDES, points out, “if today we have a company capable of manufacturing high-tech equipment in Brazil, it is not luck or chance. It is a collective effort of Brazilian society. That started with the Technological Institute of Aeronautics (ITA), passed through USP's laboratories, received funding from FAPESP research, gained scale with investment funds from BNDES and FINEP, obtained subsidized credit from BNDES-PSI, and now it will help Brazil to face the pandemic, strengthening the SUS” [25].

It is worth mentioning Anvisa's actions and efforts with the issuance of instruments and transitory flexibility applicable to the confrontation of COVID-19. Specific resolutions have been published to address lung ventilators. Such efforts have contributed to expanding the availability of lung ventilators with accurate health records. In June 2020, 70 records of pressure and volume lung ventilators were located.

In consultation with CNES, in December 2020, the installed capacity of lung ventilators in SUS also increased considerably, from 43,758 units in USE in SUS to 60,089, Table 4.

**Table 4:** Updated CNES installed capacity

**Code**	**Equipment**	**Existing**	**In Use**	**Existing SUS**	**In Use SUS**
64	Lung Ventilators	84,048	79,874	63,524	60,089

Source: extracted from CNES-DATASUS http://cnes2.datasus.gov.br/. Elaboration of the authors

## Discussion

The pandemic of the new coronavirus highlighted the weaknesses in the databases for managing health technologies in the Ministry of Health. As demonstrated, there have historically been efforts for management systems for technology investments, appropriately for the decentralization of resources with a view of the institution's acquisitions. However, there are still no tools to assist the management of these post-acquisition technologies. CNES data, at the beginning of the pandemic, suggested that about 3,000 lung ventilators were out of use, and it is not possible to identify the reasons for this equipment not being in use. It should be noted that the MoH systems do not have standardization of item nomenclature; they are not interoperable and connected, which makes data collection and management difficult.

The management of technologies involves, in addition to other system and infrastructure resources, trained human resources in operation and maintenance of this equipment in health establishments. The annual demand in recent years, through the approval of investment proposals from the Ministry of Health, averaged 1,800 lung ventilators per year. Given the massive centralized purchases, about 15,000 lung ventilators, efforts to adapt the management of these technologies in health services are imperative.

The analysis of the technical specifications of the lung ventilators approved in 2019, about 76% with the same specification, indicates opportunities for using government purchasing powers through possible centralized purchases with the possibility of more significant advantage and economy, especially to establish maintenance clauses and post-investment data.

In less than a year, the Ministry of Health acquired several lung ventilators, 8.55 times greater than the annual average of equipment acquired from 2016 to 2019. It should be noted that, to date, there has been no maintenance or management plan or strategy for this equipment, especially in a post-pandemic scenario. It should be noted that according to PNGTS, it is up to the MoH to support managers in the implementation of technologies and in their monitoring and maintenances after incorporation.

## Conclusion

Technology management lacks interoperable data and systems for its implementation. Data fragmentation, absence of technology databases compromises public policies and weakens the performance of health services.

Furthermore, the articulation of public policies that combine efforts to ensure the population's access to the SUS is crucial. The interruption of the activities of the Industrial Health Complex, especially of the GECIS meetings, in the year preceding the pandemic, demonstrates the fragility of the continuity of government efforts in the medium and long term for national productive development, with a reduction in the dependencies of the international market.

Therefore, based on the themes discussed here, it is possible to conclude that the Ministry of Health needs to improve health technology management systems. On the scale of the Policy, it is necessary to commit to permanent and long-term actions to ensure sustainability and reduce the technological vulnerabilities of the SUS.

## Data Availability

Search results can be made available upon request to the corresponding author.
